# Three-Dimensional Nanoscale Morphological Surface Analysis of Polymeric Particles Containing *Allium sativum* Essential Oil

**DOI:** 10.3390/ma15072635

**Published:** 2022-04-03

**Authors:** Larissa Medeiros de Oliveira, Robert Saraiva Matos, Ştefan Ţălu, Ana Luisa Farias Rocha, Ronald Zico de Aguiar Nunes, Jaqueline de Araújo Bezerra, Pedro Henrique Campelo Felix, Natália Mayumi Inada, Edgar Aparecido Sanches, Henrique Duarte da Fonseca Filho

**Affiliations:** 1 Laboratory of Nanostructured Polymers (NANOPOL—@nanopol_ufam), Department of Physics, Federal University of Amazonas (UFAM), Manaus 69067-005, AM, Brazil; larissa.medeiros.oli@gmail.com (L.M.d.O.); luisa14ana99@gmail.com (A.L.F.R.); ronaldzico14@gmail.com (R.Z.d.A.N.); sanchesufam@ufam.edu.br (E.A.S.); 2 Amazonian Materials Group, Department of Physics, Federal University of Amapá (UNIFAP), Macapá 68903-419, AP, Brazil; amazonianmaterialsgroup@gmail.com; 3 Graduate Program in Materials Science and Engineering, Department of Materials Science and Engineering, Federal University of Sergipe (UFS), São Cristóvão 49100-000, SE, Brazil; 4 The Directorate of Research, Development and Innovation Management (DMCDI), Technical University of Cluj-Napoca, 15 Constantin Daicoviciu St., 400020 Cluj-Napoca, Romania; 5 Federal Institute of Education, Science and Technology of Amazonas (IFAM), IFAM Analytical Center, Manaus Centro Campus, Manaus 69067-005, AM, Brazil; jaqueline.araujo@ifam.edu.br; 6 School of Agrarian Science, Federal University of Amazonas (UFAM), Manaus 69067-005, AM, Brazil; pcampelo.felix@gmail.com; 7 São Carlos Institute of Physics (IFSC), University of São Paulo (USP), São Carlos 13566-590, SP, Brazil; nataliainada@ursa.ifsc.usp.br; 8 Laboratory of Synthesis of Nanomaterials and Nanoscopy (LSNN), Department of Physics, Federal University of Amazonas (UFAM), Manaus 69067-005, AM, Brazil

**Keywords:** morphological parameters, atomic force microscopy, biodegradable particles, *Allium sativum*, essential oil

## Abstract

Biodegradable particles were developed using poly-ε-caprolactone and gelatin carriers containing different concentrations of *Allium sativum* essential oil (EO) (360 µg/mL, 420 µg/mL, and 460 µg/mL). Atomic force microscopy was useful to evaluate the particles’ surface based on morphological parameters. The particles’ size varied from 150 nm to 300 nm. The diameter was related to the increase of the particles’ height as a function of the EO concentration, influencing the roughness of the surface core values (from 20 to 30 nm) and surface irregularity. The spatial parameters Str (texture aspect ratio) and Std (texture direction) revealed low spatial frequency components. The hybrid parameters Sdq (root mean square gradient) and Sdr (interfacial area ratio) also increased as a function of the EO concentration, revealing fewer flat particles. On the other hand, the functional parameters (inverse areal material ratio and peak extreme height) suggested differences in surface irregularities. Higher concentrations of EO resulted in greater microtexture asperity on the particles’ surface, as well as sharper peaks. The nanoscale morphological surface analysis allowed the determination of the most appropriate concentration of encapsulated EO, influencing statistical surface parameters.

## 1. Introduction

*Allium sativum* bulbs (Asparagale: Amaryllidaceae) are well known worldwide due to their health benefits, as well as culinary applications [1]. The antimicrobial [2], antifungal [3,4], and antioxidant [1] bioactivity of this essential oil has been extensively reported in the scientific literature. Organosulfur and phenolic compounds are the major essential oil compounds [5,6]. The insertion of nanotechnology in food products such as gelatin to develop alternative controlling agents represents an interesting area of research [7,8,9]. Encapsulation techniques increase the stability of EO in processing and storage, as well as reduce their natural volatility, oxidation, and photodegradation [10]. Furthermore, the encapsulation process allows their controlled release [11,12]. The emulsion–diffusion encapsulation technique enables particle size control, good reproducibility, and high encapsulation efficiency [13,14,15,16,17,18,19]. In this work, different concentrations of *A. sativum* essential oil were encapsulated in particles constituted of poly-ε-caprolactone (PCL) and gelatin carriers.

The development of controlled release systems requires a systematic understanding of the particles’ surface. The atomic force microscopy (AFM) technique has been extensively applied to evaluate complex surfaces [20,21,22,23], providing information on morphological [24,25,26,27] and fractal [28,29,30] parameters, as well as a power spectrum density (PSD) [31,32,33,34]. These parameters allow the proposition of specific applications associated with the release mechanism of encapsulated compounds [35]. There are several free and commercial softwares to perform morphological evaluations, representing useful alternative tools for material surface characterization.

The aim of this paper was to evaluate the morphology and spatial frequency (to understand the dynamics of topographic changes) of the PCL/gelatin particles to identify surface patterns of interest for technological purposes. 

A novelty discussion of statistical surface parameters related to nanoparticle-based polymeric carriers was reported here based on the AFM technique and the use of image processing. Our results allowed us to understand the influence of various essential oil concentrations on the evaluated statistical surface parameters, such as roughness, volume, peak distribution, height distribution and texture homogeneity, providing many important aspects of polymeric films surface, including the capsules’ shape. All analyses were performed using standards such as the International Standardization Organization (ISO).

## 2. Materials and Methods

### 2.1. Materials

All aqueous solutions were prepared using ultrapure deionized water (18.2 M.Ω.cm, Gehaka, Master WFI, São Paulo, SP, Brazil). Dichloromethane and acetone were purchased from LabSynth, Diadema, SP, Brazil. Gelatin, Tween 80^®^, PCL, sorbitan stearate (Span 60), and caprylic/capric acid triglyceride (TAAC) were purchased form Sigma-Aldrich, Barueri, Brazil. Transglutaminase was purchased from GastronomyLab, Brasília, Brazil.

### 2.2. Essential Oil Encapsulation

*A. sativum* bulbs were collected in Manaus/AM, Brazil, and dried under controlled humidity at 30 °C until reaching constant weight. Then, 400 g of milled bulbs were subjected to hydrodistillation using a Clevenger-type apparatus (NetLab, Tatuapé, SP, Brazil) for 2 h at 100 °C. The essential oil was stored at −18 °C.

PCL/Gelatin-based particles loaded with *A. sativum* essential oil were synthesized based on previous report [36] with modifications. Solution I was prepared as follows: Gelatin was heated to 50 °C in distilled water under constant stirring. Then, Tween 80^®^ (0.30 g) was added to Solution I when the temperature decreased to 40 °C. Solution II was prepared using PCL (0.05 g), sorbitan stearate (span 60; 0.02 g), and caprylic/capric acid triglyceride (TACC; 0.1 g) solubilized in acetone (15 mL). Different concentrations of essential oil (360 μg/mL (P_360_), 420 μg/mL (P_420_), or 460 μg/mL (P_460_)) were added to Solution II, which was transferred to Solution I under constant stirring (10,000 rpm) using an ultra-disperser. Transglutaminase (0.19 g) was added to the final solution. Then, the formulation was kept under constant stirring until acetone evaporation. All systems were maintained in bio-oxygen demand (BOD) chamber at 25 °C in sealed vials. P_0_ represents the unloaded system.

### 2.3. AFM Imaging

The systems NP0, NP360, NP420, and NP460 94 (1 μL) were dripped on a glass slide and, to speed up the drying process, we exposed the films to a flow of nitrogen gas with the aid of an air gun to control this flow on the surface. Then, the glass slide containing the formed film was fixed on the AFM sample holder using an adhesive tape. Measurements were performed at room temperature (296 ± 1 K) and (40 ± 1)% R.H. on an Innova (Bruker^®^, Billerica, MA, USA) equipment operated in tapping mode and equipped with a silicon tip and Al coated cantilever with a spring constant of 42 N/m (Tap190AL-G from Budget SensorsTM, Izgrev, Sofia, Bulgária). Scans were performed using (10 × 10) µm^2^ with (256 × 256) pixels at a scan rate of 1 Hz.

### 2.4. Surface Analysis

The AFM images were analyzed using the Mountains Map^®^ (Besançon, France), Premium software version 8.4.8872 [37]. Morphological parameters were extracted according to the ISO 25178-2: 2012 [38]. Height, functional, spatial, hybrid, feature, core roughness Sk, and volume parameters were discussed according to their previous definition [24,39,40,41]. Additionally, the one-dimensional power spectrum density (PSD) was evaluated using the free software WsxM© 5.0 (Nanotec Electrónica S.L., Madrid, Spain) [42] using the height distribution autocorrelation function from Equation (1) [43], where *L_z_* is the image pixels number per line and *q_z_* is the vector associated with the *z* value of the height distribution. The average PSD was obtained from the topographic maps using Equation (2) [43].

(1) $$h\left(x,y\right)=L_{z}{}^{-1}\sum_{q_{z}}PSD^{1D}\left(q_{z}\right)\;e^{iq_{z}z},$$

(2) $$PSD^{1D}\left(q_{x}\right)=L_{x}{}^{-1}{\left[\int_{L_{x}}h\left(x,y\right)e^{-iq_{x}x}\right]}^{2},$$

The linearized graph (PSD x q) was obtained from the spectrum data using Equation (3), where C_0_ is constant.

PSD = C_0._q^−2−2H^, (3)

The Hurst coefficient (H) of the surface nanotexture was estimated from the linearized graph using Equation (4) [43,44], where *µ* is the absolute slope of the average PSD curve.

(4) $$Hc=\frac{µ-2}{2},$$

### 2.5. Statistical Analysis

Statistical analysis was performed using the OriginPro^®^ software version 9.0 (OriginLab Corporation, Northampton, MA, USA). The variance analysis (ANOVA) was applied using a 5% significance level (*p*-value < 0.05) based on Tukey’s test.

## 3. Results

### 3.1. Morphological Analysis

The AFM technique allows an accurate surface analysis, providing a significant increase of spatial resolution compared to all other far-field microscopy optical methods. The lateral resolution is determined by the tip radius (~20 nm or less). On the other hand, the vertical resolution reaches nanometer fractions, allowing a significant evaluation of the particle’s height and surface roughness. For this reason, the morphological evaluation of the developed particles as a function of the essential oil concentration was investigated here using this powerful technique.

**Figure 1:**
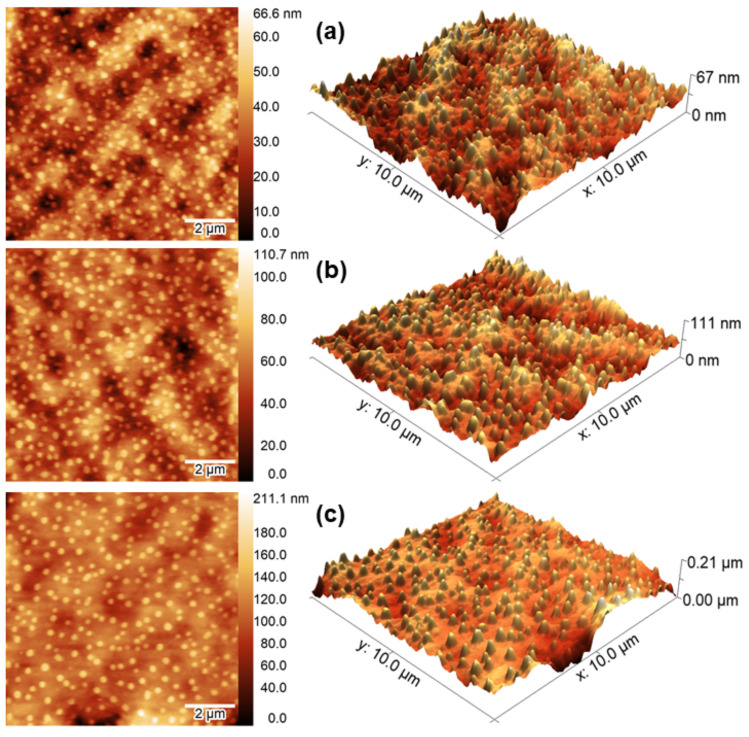
Two-dimensional and 3D AFM images of (**a**) P_360_, (**b**) P_420_, and (**c**) P_460_ particle surfaces.

Figure 1 shows the 2D and 3D AFM topographical maps. Flat regions on the particle’s surface should be preferred due to the intrinsic features of this technique. For this reason, particles presenting curved surfaces can limit the reliability of the morphometric data and restrict the investigation of samples presenting marginal complex three-dimensional shell configuration [45]. The particles’ topography evaluated here was dependent on the fashion they were dripped on the glass surface, as well as on the drying process, so irregularities were expected. For this reason, smaller scans were performed to overcome this problem. The average particle diameter, height, and height/diameter ratio are shown in Table 1 [46].

The particles developed had spherical cap-like shapes and showed a uniform size distribution. The average size varied from 184 ± 30 nm to 261 ± 47 nm. The increase in the average diameter was accompanied by the average increase in the height of the particles, but not in the same proportion (since the concentration of essential oil also increased), as shown in Table 1. Due to significant differences in the height values, the height/diameter ratios were obtained: a significant increase was observed as a function of the encapsulated essential oil concentration. Other reports have applied the AFM technique to estimate the particles range for systems formed by gelatin and PCL [35,36,47].

### 3.2. Stereometric Evaluation Evaluation

Statistical stereometric evaluation parameters are shown in Table 2. Most of these parameters presented a significant difference (*p*-value < 0.05), except the area material ratio (Smr), texture aspect ratio (Str), and texture direction (Std).

From the functional parameter values, only the Smr parameter was similar in all developed systems, exhibiting the highest possible proportion (100%) [40]. However, the inverse areal material ratio (Smc) and the peak extreme height (Sxp) values increased as a function of the essential oil concentration. From the medium plane to the highest peak, the proportion of material increased for higher essential oil concentrations, suggesting differences in surface irregularities.

Considering the spatial parameters, the Str and Std average values were not significant, revealing similar isotropy patterns. Furthermore, the autocorrelation length (Sal) parameter decreased from P_360_ to P_460_. This result shows that the developed systems are dominated by low spatial frequency components [24], providing patterns with relatively irregular texture, as more regular textures present *S_al_* ≤ 0.2 [40].

Considering the hybrid parameters, the root mean square gradient (Sdq) and developed interfacial area ratio (Sdr) increased when the concentration of EO was increased. Less flat topographical spatial patterns were observed when the concentration of EO was increased (full flat surface present Sdq = 0 and Sdr = 0% [24,40]).

However, considering the feature parameters, a slight decrease of the peak density (Spd) was observed because this parameter decreased when the concentration of EO was increased. This result indicated greater spacing between rough peaks for high EO concentrations. Moreover, the peak shape also became more pointed when the concentration of EO was increased (as the arithmetic mean peak curvature (Spc) increased from P_360_ to P_460_), suggesting a growth of peaks.

### 3.3. Watershed Segmentation of Particles’ Microtexture

**Figure 2:**
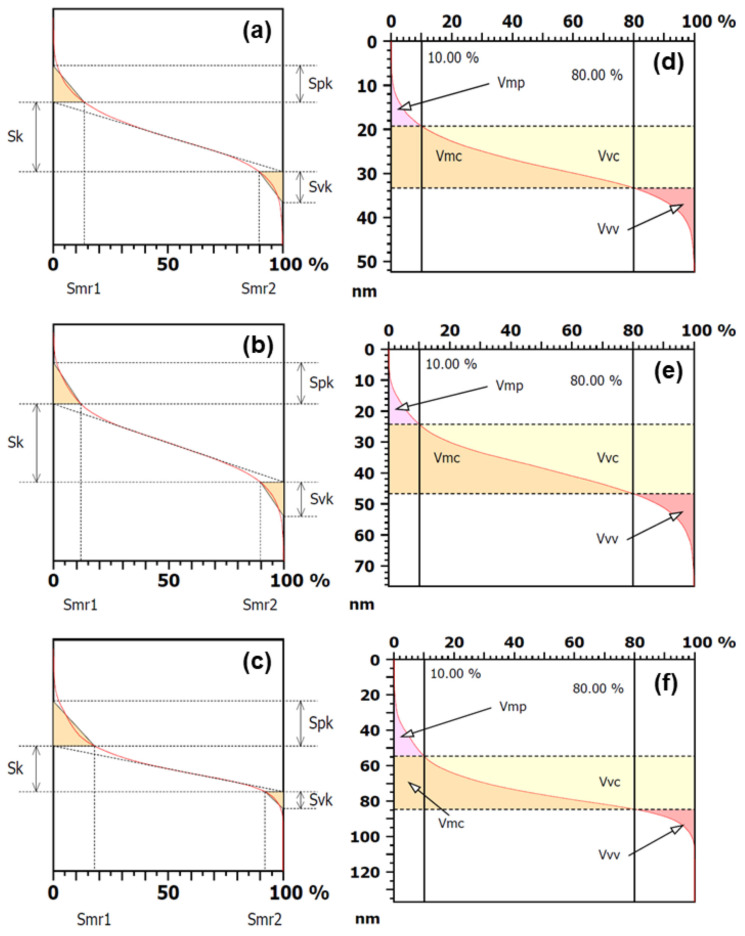
Graphical evaluation of Sk parameters (**left**) and volume parameters (**right**) based on the Abbott curve calculated for (**a**,**d**) P_360_, (**b**,**e**) P_420_, and (**c**,**f**) P_460_.

The thickness and volume parameters related to the core of the surface’s microtexture are shown in Table 3, and their representations are shown in Figure 2. All parameters presented a significant difference (*p*-value < 0.05), except the valley material portion (Smr2) parameter. The roughness of the surface’s core increased because the parameters core roughness Sk, reduced peak height (Spk), and reduced valley depth (Svk) increased as a function of the EO concentration. For this reason, the roughness of the surface’s core was increased, resulting in a significant surface irregularity. Furthermore, the peak material portion (Smr1) parameter decreased from P_360_ to P_460_. This result was consistent, although the Smr2 parameter presented a similar value in all developed particles. Similarly, the parameters Sk, peak material volume (Vmp), core material volume (Vmc), dale void volume (Vvc), and core void volume (Vvv) increased from P_360_ to P_460_. This result reveals that the volume of the material that can be deposited on the peaks and valleys of the surface increased as a function of the essential oil concentration. For this reason, physical properties such as friction, wear, and adhesion can be affected. This result is a consequence of the increase in global and local roughness as the increase of particles (diameter and height, as shown in Table 1) promoted an increase of roughness in all surface regions.

### 3.4. Power Spectrum Density (PSD) of the Surface Nanotexture

**Figure 3:**
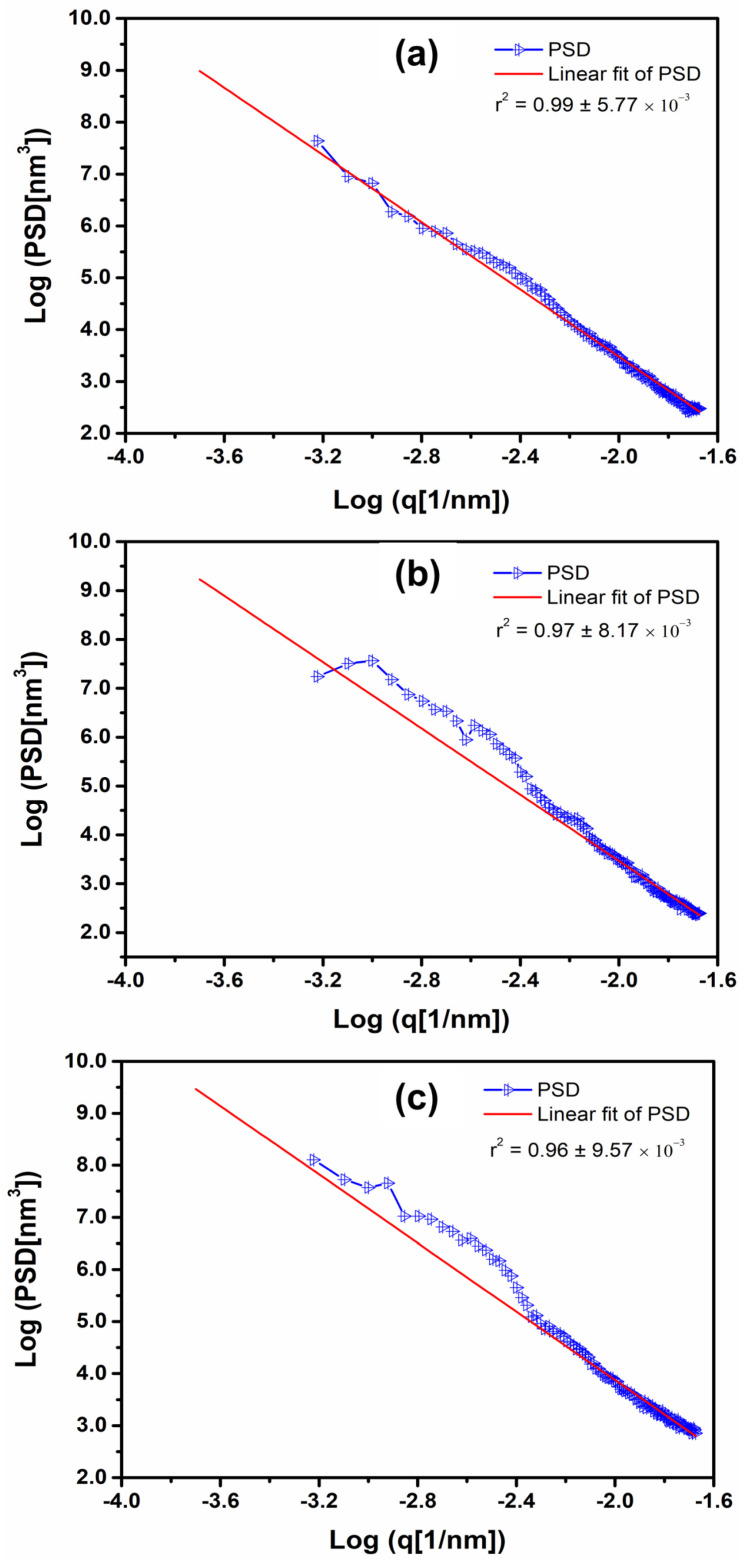
Average PSD spectrum of (**a**) P_360_, (**b**) P_420_, and (**c**) P_460_ systems.

The power spectrum density (PSD) evaluation has been extensively used to define different patterns in several systems [31,32,33,34]. The average PSD spectrum of the developed particles is shown in Figure 3, revealing a small difference in the spatial complexity of the particles, mainly in the P_360_ and P_420_ systems. A statistical difference (*p*-value < 0.05) was observed.

According to the Hurst (H) results, the P_360_ system was controlled by higher spatial frequencies, resulting in (0.59 ± 0.04), (0.70 ± 0.04), and (0.065 ± 0.02), respectively, for the systems P_360_, P_420,_ and P_460_. Higher concentrations of essential oil promoted a decrease of the dominant spatial frequencies of the particle surfaces. These results agree with the reported Sal values (P_360_ presented spatial dominant frequencies greater than P_420_ and P_460_). Furthermore, as the Tukey test revealed that the P_420_ and P_460_ systems are similar, a significant morphology modification was better noted in P_360_ to P_420_ (also in agreement with the obtained height parameters).

## 4. Conclusions

A systematic 3D nanoscale morphological surface analysis of polymeric particles containing *Allium sativum* essential oil was performed. The developed particles presented spherical cap-like shapes and, depending on the EO concentration, their average sizes ranged from 184 ± 30 nm to 261 ± 47 nm. The increase of the average diameter occurred as the concentration of EO was also increased. Based on the Hurst (H) results, the values of the H parameter were found around (0.59 ± 0.04), (0.70 ± 0.04), and (0.065 ± 0.02), respectively, for the systems P_360_, P_420_ and P_460_, indicating the P_360_ system was controlled by higher spatial frequencies. Therefore, higher concentrations of EO promoted a decrease of the dominant spatial frequencies of the particle surfaces, which agrees with the Sal-reported values. The evaluation of those stereometric parameters, especially functional, spatial, hybrid, and feature parameters, can influence the development of particles with suitable surface properties for technological application. According to our results, we can suggest that the P_420_ and P_460_ systems presented better conditions for application as biodefensive, since the morphological analysis revealed greater microtexture asperity, lower density of rough peaks, and sharper peaks, which can have an influence on the suitable anchoring of the particle onto another surface and EO release.

## Figures and Tables

**Table 1:** Average particle diameter, height, and height/diameter ratio.

Parameter	P_360_	P_420_	P_460_
Diameter (nm)	184 ± 30	236 ± 49	261 ± 47
Height (nm)	18 ± 5	31 ± 6	56 ± 6
Height/Diameter (nm)	0.09 ± 0.04	0.13 ± 0.05	0.21 ± 0.06

**Table 2:** Statistical morphological parameters according to ISO 25178-2: 2012.

Parameter	P_360_	P_420_	P_460_
**Functional**			
Smr * (%)	100 ± 0	100 ± 0	100 ± 0
Smc (nm)	10 ± 1	15 ± 1	21 ± 1
Sxp (nm)	15 ± 2	20.4 ± 0.3	24 ± 3
**Spatial**			
Sal (nm)	0.7 ± 0,1	0.64 ± 0.07	0.49 ± 0.06
Str *	0.49 ± 0.06	0.37 ± 0.07	0.6 ± 0.2
Std * (°)	131 ± 74	132 ± 74	133 ± 74
**Hybrid**			
Sdq (-)	0.039 ± 0.001	0.064 ± 0.001	0.100 ± 0.004
Sdr (%)	0.076 ± 0.005	0.22 ± 0.02	0.49 ± 0.04
**Feature**			
Spd (1/μm²)	1.2 ± 0.1	1.14 ± 0.04	0.92 ± 0.06
Spc (1/μm)	0.67 ± 0.05	1.11 ± 0.07	2.3 ± 0.3

* Samples without significant difference ANOVA One-Way (*p* < 0.05).

**Table 3:** *S_k_* and volume parameters according to ISO 25178-2:2012.

Parameter	P_360_	P_420_	P_460_
Sk (nm)	20 ± 2	29.013 ± 3	30.484 ± 4
Spk (nm)	8.9 ± 0.9	13.397 ± 0.828	26.031 ± 4
Svk (nm)	7.2 ± 0.8	9 ± 1	14 ± 2
Smr1 (%)	13 ± 1	12.0 ± 0.7	17 ± 1
Smr2 * (%)	89.7 ± 0.8	92 ± 1	91.0 ± 0.9
Vmp (µm^3^/µm^2^)	4 × 10 ^–4^ ± 4 × 10^–^^5^	6 × 10^–^^4^ ± 4 × 10^–^^5^	1 × 10^–^^3^ ± 1 × 10^–^^4^
Vmc (µm^3^/µm^2^)	7 × 10^–^^3^ ± 9 × 10^–^^4^	1.02 × 10^–^^2^ ± 8.18 × 10^–^^4^	1.19 × 10^–^^2^ ± 1.04 × 10^–^^3^
Vvc (µm^3^/µm^2^)	1 × 10^–^^2^ ± 1 × 10^–^^3^	2 × 10^–^^2^ ± 1 × 10^–^^4^	2 × 10^–^^2^ ± 1 × 10^–^^3^
Vvv (µm^3^/µm^2^)	9 × 10^–^^4^ ± 7 × 10^–^^5^	1 × 10^–^^3^ ± 7 × 10^–^^4^	1 × 10^–^^3^ ± 2 × 10^–^^4^

* Samples without significant difference ANOVA One-Way (*p* < 0.05).

## Data Availability

Not applicable.

## References

[1] Lawrence R., Lawrence K. (2011). Antioxidant activity of garlic essential oil (*Allium Sativum*) grown in north Indian plains. Asian Pac. J. Trop. Biomed..

[2] Araújo M.K., Gumiela A.M., Bordin K., Luciano F.B., de Macedo R.E.F. (2018). Combination of garlic essential oil, allyl isothiocyanate, and nisin Z as bio-preservatives in fresh sausage. Meat Sci..

[3] Matan N., Matan N., Ketsa S. (2012). Effect of heat curing on antifungal activities of anise oil and garlic oil against *Aspergillus niger* on rubberwood. Int. Biodeterior. Biodegrad..

[4] Long Y., Huang W., Wang Q., Yang G. (2020). Green synthesis of garlic oil nanoemulsion using ultrasonication technique and its mechanism of antifungal action against *Penicillium italicum*. Ultrason. Sonochem..

[5] Visani V., Netto J.M.S., Honorato R.S., de Araújo M.C.U., Honorato F.A. (2017). Screening analysis of garlic-oil capsules by infrared spectroscopy and chemometrics. Microchem. J..

[6] Ragavan G., Muralidaran Y., Sridharan B., Ganesh R.N., Viswanathan P. (2017). Evaluation of garlic oil in nano-emulsified form: Optimization and its efficacy in high-fat diet induced dyslipidemia in Wistar rats. Food Chem. Toxicol..

[7] Al-Tayyar N.A., Youssef A.M., Al-Hindi R.R. (2020). Edible coatings and antimicrobial nanoemulsions for enhancing shelf life and reducing foodborne pathogens of fruits and vegetables: A review. Sustain. Mater. Technol..

[8] El-Sayed S.M., El-Sayed H.S., Ibrahim O.A., Youssef A.M. (2020). Rational design of chitosan/guar gum/zinc oxide bionanocomposites based on *Roselle calyx* extract for Ras cheese coating. Carbohydr. Polym..

[9] Moustafa H., El-Sayed S.M., Youssef A.M. (2021). Synergistic impact of cumin essential oil on enhancing of UV-blocking and antibacterial activity of biodegradable poly(butylene adipate-co-terephthalate)/clay platelets nanocomposites. J. Thermoplast. Compos. Mater..

[10] Piletti R., Zanetti M., Jung G., de Mello J.M.M., Dalcanton F., Soares C., Riella H.G., Fiori M.A. (2019). Microencapsulation of garlic oil by β-cyclodextrin as a thermal protection method for antibacterial action. Mater. Sci. Eng. C..

[11] Vahedikia N., Garavand F., Tajeddin B., Cacciotti I., Jafari S.M., Omidi T., Zahedi Z. (2019). Biodegradable zein film composites reinforced with chitosan nanoparticles and cinnamon essential oil: Physical, mechanical, structural and antimicrobial attributes. Colloids Surf. B Biointerf..

[12] Hadidi M., Pouramin S., Adinepour F., Haghani S., Jafari S.M. (2020). Chitosan nanoparticles loaded with clove essential oil: Characterization, antioxidant and antibacterial activities. Carbohydr. Polym..

[13] Hosseini S.F., Zandi M., Rezaei M., Farahmandghavi F. (2013). Two-step method for encapsulation of oregano essential oil in chitosan nanoparticles: Preparation, characterization and in vitro release study. Carbohydr. Polym..

[14] Lin Q., Ji N., Li M., Dai L., Xu X., Xiong L., Sun Q. (2020). Fabrication of debranched starch nanoparticles via reverse emulsification for improvement of functional properties of corn starch films. Food Hydrocoll..

[15] El-Sayed S.M., El-Sayed H.S. (2021). Antimicrobial nanoemulsion formulation based on thyme (*Thymus vulgaris*) essential oil for UF labneh preservation. J. Mater. Res. Technol..

[16] Saada N.S., Abdel-Maksoud G., El-Aziz M.S.A., Youssef A.M. (2020). Evaluation and utilization of lemongrass oil nanoemulsion for disinfection of documentary heritage based on parchment. Biocatal. Agric. Biotechnol..

[17] Calderó G., Montes R., Llinàs M., García-Celma M.J., Porras M., Solans C. (2016). Studies on the formation of polymeric nano-emulsions obtained via low-energy emulsification and their use as templates for drug delivery nanoparticle dispersions. Colloids Surf. B Biointerf..

[18] Moinard-Checot D., Chevalier Y., Briançon S., Fessi H., Guinebretière S. (2006). Nanoparticles for Drug Delivery: Review of the formulation and process difficulties illustrated by the emulsion-diffusion process. J. Nanosci. Nanotechnol..

[19] Solans C., Izquierdo P., Nolla J., Azemar N., Garciacelma M. (2005). Nano-emulsions. Curr. Opin. Colloid Interface Sci..

[20] Ferraro M.A.N., Pinto E.P., Matos R.S. (2020). Study of the superficial distribution of microorganisms in kefir biofilms prepared with Cupuaçu juice. J. Bioenergy Food Sci..

[21] Almeida P.A., Pinto E.P., Filho H.D.F., Matos R.S. (2019). Distribution of microorganisms on surface of Kefir biofilms associated with Açaí extract. Sci. Amaz..

[22] Matos T.J.R., Ramos G.Q., Matos R.S., da Fonseca Filhio H.D. (2019). Medição da área foliar de *Anacardium occidentale* L. baseada em processamento digital de imagens. Sci. Amaz..

[23] Ramos G.Q., Matos R.S., da Fonseca Filho H.D. (2020). Advanced microtexture study of *Anacardium occidentale* L. leaf surface from the Amazon by fractal theory. Microsc. Microanal..

[24] Matos R.S., Pinto E.P., Ramos G.Q., da Fonseca de Albuquerque M.D., da Fonseca Filho H.D. (2020). Stereometric characterization of kefir microbial films associated with *Maytenus rigida* extract. Microsc. Res. Tech..

[25] Méndez A., Reyes Y., Trejo G., Stępień K., Ţălu Ş. (2015). Micromorphological characterization of zinc/silver particle composite coatings. Microsc. Res. Technol..

[26] Ţălu Ş., Stach S., Zaharieva J., Milanova M., Todorovsky D., Giovanzana S. (2014). Surface roughness characterization of poly(methylmethacrylate) films with immobilized Eu(III) β-Diketonates by fractal analysis. Int. J. Polym. Anal. Charact..

[27] Ţălu Ş., Matos R.S., Pinto E.P., Rezaee S., Mardani M. (2020). Stereometric and fractal analysis of sputtered Ag-Cu thin films. Surf. Interf..

[28] Ţălu Ş., Abdolghaderi S., Pinto E.P., Matos R.S., Salerno M. (2020). Advanced fractal analysis of nanoscale topography of Ag/DLC composite synthesized by RF-PECVD. Surf. Eng..

[29] Omar M., Salcedo C., Ronald R., Zamora M., Tavares C. (2016). Study fractal leaf surface of the plant species *Copaifera sp*. using the Microscope Atomic-Force-AFM, Rev. ECIPerú.

[30] Matos R.S., Ramos G.Q., da Fonseca Filho H.D., Ţălu Ş. (2020). Advanced micromorphology study of microbial films grown on Kefir loaded with Açaí extract. Micron.

[31] Senthilkumar M., Sahoo N.K., Thakur S., Tokas R.B. (2005). Characterization of microroughness parameters in gadolinium oxide thin films: A study based on extended power spectral density analyses. Appl. Surf. Sci..

[32] Barcelay Y.R., Moreira J.A.G., Almeida A.D.J.M., Brito W.R., Matos R.S., da Fonseca Filho H.D. (2020). Nanoscale stereometric evaluation of BiZn_0.5_Ti_0.5_O_3_ thin films grown by RF magnetron sputtering. Mater. Lett..

[33] Arman A., Ţălu Ş., Luna C., Ahmadpourian A., Naseri M., Molamohammadi M. (2015). Micromorphology characterization of copper thin films by AFM and fractal analysis. J. Mater. Sci. Mater. Electron..

[34] Gong Y., Misture S.T., Gao P., Mellott N.P. (2016). Surface roughness measurements using Power Spectrum Density analysis with enhanced spatial correlation length. J. Phys. Chem. C.

[35] de Oliveira L.M., Silva L.S., Mar J.M., Azevedo S.G., Rabelo M.S., da Fonseca Filho H.D., Lima S.X., Bezerra J.D.A., Machado M.B., Campelo P.H. (2019). Alternative biodefensive based on the essential oil from *Allium sativum* encapsulated in PCL/Gelatin Nanoparticles. J. Food Eng. Technol..

[36] Silva L.S., Mar J.M., Azevedo S.G., Rabelo M.S., Bezerra J.A., Campelo P.H., Machado M.B., Trovati G., Santos A.L.d., da Fonseca Filho H.D. (2019). Encapsulation of *Piper aduncum* and *Piper hispidinervum* essential oils in gelatin nanoparticles: A possible sustainable control tool of *Aedes aegypti*, *Tetranychus urticae* and *Cerataphis lataniae*. J. Sci. Food Agric..

[37] Mountains Map® 8 Premium Software (Digital Surf, Besançon, France). http://www.digitalsurf.fr.

[38] ISO 2012 ISO 25178-2:2012 - Geometrical Product Specifications (GPS)—Surface Texture: Areal—Part 2: Terms, Definitions and Surface Texture Parameters. https://www.iso.org/standard/42785.html.

[39] Blateyron F. (2013). The Areal Field Parameters. Characterisation Areal Surface Texture.

[40] Leach R. (2013). Characterisation of Areal Surface Texture.

[41] Franco L.A., Sinatora A. (2015). 3D surface parameters (ISO 25178-2): Actual meaning of Spk and its relationship to Vmp. Precis. Eng..

[42] Horcas I., Fernández R., Gómez-Rodríguez J.M., Colchero J., Gómez-Herrero J., Baro A.M. (2007). WSXM: A software for scanning probe microscopy and a tool for nanotechnology. Rev. Sci. Instrum..

[43] Jacobs T.D.B., Junge T., Pastewka L. (2017). Quantitative characterization of surface topography using spectral analysis. Surf. Topogr. Metrol. Prop..

[44] Martínez J.G., Nieto-Carvajal I., Abad J., Colchero J. (2012). Nanoscale measurement of the power spectral density of surface roughness: How to solve a difficult experimental challenge. Nanoscale Res. Lett..

[45] Klapetek P., Nečas D., Campbellová A., Yacoot A., Koenders L. (2011). Methods for determining and processing 3D errors and uncertainties for AFM data analysis. Meas. Sci. Technol..

[46] Nečas D., Klapetek P. (2012). Gwyddion: An open-source software for SPM data analysis. Cent. Eur. J. Phys..

[47] de Oliveira L.M., Lima S.X., Silva L.S., Mar J.M., Azevedo S.G., Rabelo M.S., Henrique D., Filho F., Campelo P.H., Sanches E.A. (2019). Controlled release of *Licaria puchury-major* essential oil encapsulated in PCL/gelatin-based colloidal systems and membranes. Am. J. Essent. Oils Nat. Prod..

